# eNOS expression in umbilical cord arterial endothelial cells depends on placental perfusion and is triggered by adaptive chromatin signatures

**DOI:** 10.1186/1756-8935-6-S1-P31

**Published:** 2013-03-18

**Authors:** Jan Postberg, Miriam Kanders, Valerie Orth, Stefan Wirth, Andreas Jenke

**Affiliations:** 1 Zentrum für Kinder- und Jugendmedizin, HELIOS Klinikum Wuppertal, Universität Witten/Herdecke, Wuppertal, Germany

## Background

To contribute to our limited knowledge about placental insufficiency and consecutive dystrophy, identification of adaptive molecular mechanisms applied by the fetus might be helpful.

## Methods

Epidemiological parameters, Doppler examinations as well as DNA/RNA samples purified from endothelial umbilical artery cells of newborns were collected during a 6 month period. Expression of eNOS was quantified using qPCR. As putative adaptive epigenomic signatures involved in gene regulation 5meC/5hmeC levels as well as histone post-translational modification patterns of the eNOS promoter were analyzed using (h)MeDIP-qPCR and ChIP-qPCR, respectively. Moreover genotyping of a putative miRNA locus was performed.

## Results

42 patients were included in this study (gestational age range 23 + 6 to 41+7, median 31+3). eNOS copy numbers were significantly higher in infants with impaired perfusion in the uterine and mediocerebral artery (mean 15.7 vs. 228.6). The eNOS promoter region was hypomethylated at the transcription start site in all patients and no significant correlation with gene expression levels could be observed. Also there was no association between intronic 27-nt miRNA genotype status and eNOS expression. However histone H3 acetylation was significantly higher in patients with impaired placental perfusion. Treatment of human umbilical venous cells *in vitro* with either the histone acetyl transferase inhibitor C646 or the histone deacetylase inhibitor trichostatin A also significantly altered the eNOS expression profile.

## Conclusion

Endothelial expression of eNOS in umbilical arteries can possibly be an adaptive responsive mechanism to impaired relative nutritional supply or placental perfusion. Regulation of eNOS expression seems to be independent of differential promoter 5meC/5hmeC levels or intronic miRNA genotype. Adaptive eNOS regulation apparently depends on differential posttranslational modifications of histone N-termini, such as H3ac.

